# Simple Plans or Sophisticated Habits? State, Transition and Learning Interactions in the Two-Step Task

**DOI:** 10.1371/journal.pcbi.1004648

**Published:** 2015-12-11

**Authors:** Thomas Akam, Rui Costa, Peter Dayan

**Affiliations:** 1 Champalimaud Neuroscience Program, Champalimaud Centre for the Unknown, Lisbon, Portugal; 2 Department of Experimental Psychology, University of Oxford, Oxford, United Kingdom; 3 Gatsby Computational Neuroscience Unit, UCL, London, United Kingdom; Brain and Spine Institute (ICM), FRANCE

## Abstract

The recently developed ‘two-step’ behavioural task promises to differentiate model-based from model-free reinforcement learning, while generating neurophysiologically-friendly decision datasets with parametric variation of decision variables. These desirable features have prompted its widespread adoption. Here, we analyse the interactions between a range of different strategies and the structure of transitions and outcomes in order to examine constraints on what can be learned from behavioural performance. The task involves a trade-off between the need for stochasticity, to allow strategies to be discriminated, and a need for determinism, so that it is worth subjects’ investment of effort to exploit the contingencies optimally. We show through simulation that under certain conditions model-free strategies can masquerade as being model-based. We first show that seemingly innocuous modifications to the task structure can induce correlations between action values at the start of the trial and the subsequent trial events in such a way that analysis based on comparing successive trials can lead to erroneous conclusions. We confirm the power of a suggested correction to the analysis that can alleviate this problem. We then consider model-free reinforcement learning strategies that exploit correlations between where rewards are obtained and which actions have high expected value. These generate behaviour that appears model-based under these, and also more sophisticated, analyses. Exploiting the full potential of the two-step task as a tool for behavioural neuroscience requires an understanding of these issues.

## Author Summary

Planning is the use of a predictive model of the consequences of actions to guide decision making. Planning plays a critical role in human behaviour, but isolating its contribution is challenging because it is complemented by control systems which learn values of actions directly from the history of reinforcement, resulting in automatized mappings from states to actions often termed habits. Our study examined a recently developed behavioural task which uses choices in a multi-step decision tree to differentiate planning from value-based control. We compared various strategies using simulations, showing a range that produce behaviour that resembles planning but in fact arises as a fixed mapping from particular sorts of states to action. These results show that when a planning problem is faced repeatedly, sophisticated automatization strategies may be developed which identify that there are in fact a limited number of relevant states of the world each with an appropriate fixed or habitual response. Understanding such strategies is important for the design and interpretation of tasks which aim to isolate the contribution of planning to behaviour. Such strategies are also of independent scientific interest as they may contribute to automatization of behaviour in complex environments.

## Introduction

Humans and other animals are thought to use a mixture of different strategies to learn to choose actions that lead to positive outcomes and prevent negative outcomes [1,2]. Much interest is currently focused on the distinction between control systems which employ model-based and (value-based) model-free reinforcement learning (RL) [3–14]. Model-based RL works by learning a predictive model of the specific consequences of actions, and planning by using this model to evaluate the different options prospectively. By contrast, model-free RL directly learns the value of actions through prediction errors, which quantify the difference in worth between actual and expected outcomes. These different strategies offer distinct advantages and disadvantages. Model-based RL is computationally costly and time consuming, because of the demands of planning many steps into the future before action. However, it can, in principle, use information efficiently, particularly in the face of a changing environment. This is because the implications that a change has for control in other parts of the environment can be evaluated immediately using the model without having to be directly experienced. Model-free RL incurs little computational cost and supports rapid action selection. However, it is statistically inefficient as it discards information about the specific outcomes of actions, and learns by propagating initially incorrect predictions from states to their sequential predecessors.

Dissociating the contributions of model-based and model-free RL to behaviour is challenging because under many circumstances, including most laboratory based reward guided decision making tasks, they are expected to produce similar behaviour. Outcome devaluation (or, more generally, revaluation) has traditionally been used as a gold-standard test to demonstrate the use of a simple forward model predicting the specific outcomes of actions [1,15,16]. In an outcome devaluation experiment, the subject is trained to perform two different actions, each of which obtains a different reward, e.g. pressing left or right levers for pellets of two different flavours. One reward is then devalued, for example by pairing it with illness in another context. The impact of this devaluation on the subjects’ propensity to press the levers is then tested in extinction, i.e., without any longer providing the outcomes. Model-based lever-pressing depends on a representation of the outcome to which the pressing leads, implying that subjects would prefer the lever associated with the non-devalued outcome. However, model-free lever-pressing is based on past experience of its utility, implying that subjects would not differentiate between the two levers. Use of two levers controls for general motivational effects of devaluation and extinction. In psychological terms, model-based behaviour is considered goal-directed, and model-free, habitual [1,17].

Research using outcome devaluation paradigms has established that learnt actions are initially specified by model-based RL, but can transition to being devaluation insensitive given extensive training under appropriate conditions [17,18]. This has been interpreted as a shift to model-free RL [4]. Distinct sets of brain regions have been identified as necessary for devaluation sensitive and devaluation insensitive behaviour [19–30], a finding that has been taken as implying that model-based and model-free RL are implemented by partially separate neural circuits.

**Fig 1:**
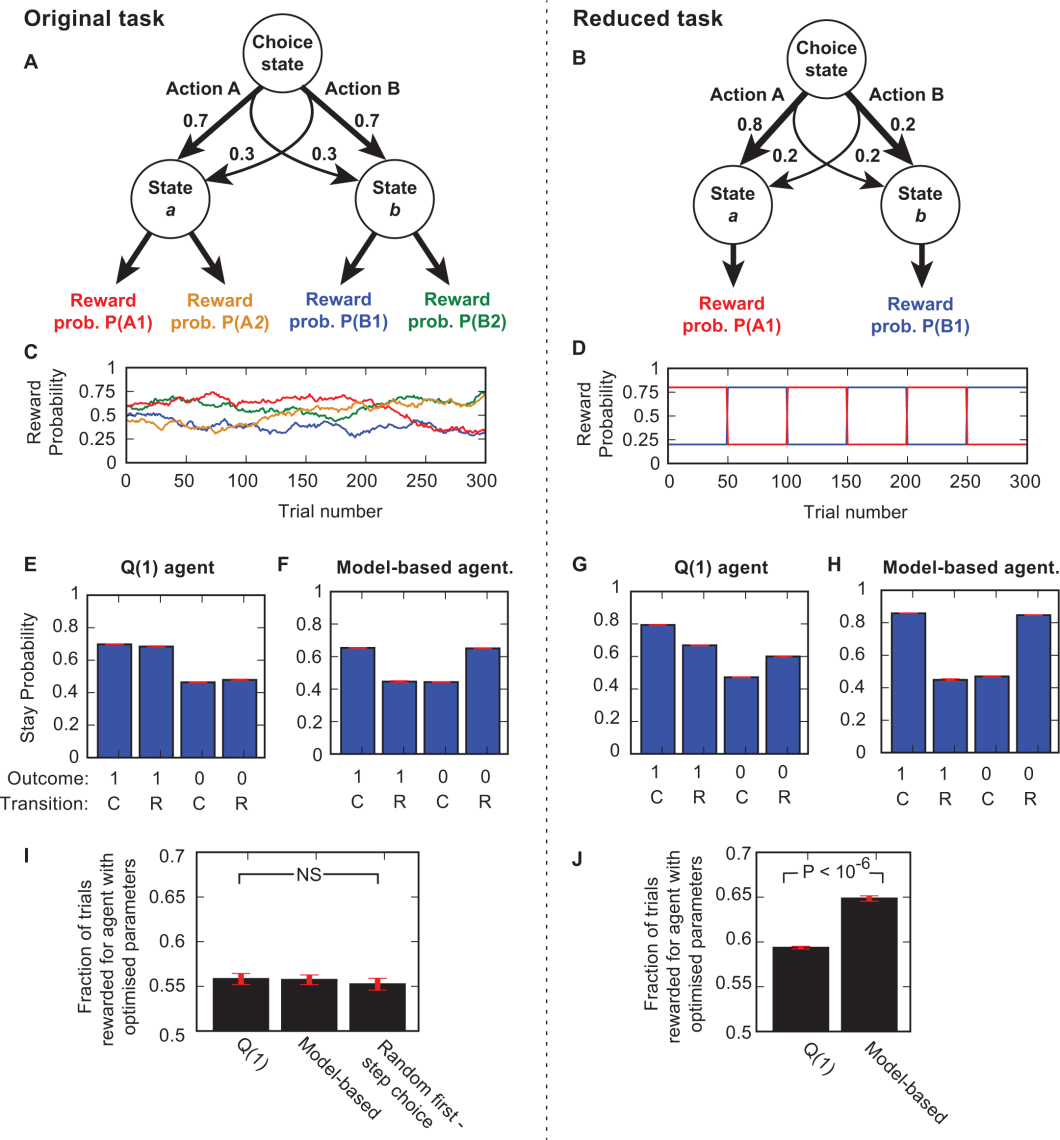
Original and reduced versions of the two-step task. (**A, B**) Diagram of task structure for original (**A**) and reduced (**B**) two step tasks. (**C**, **D**) Example reward probability trajectories for the second-step actions in each task. (**E—H**) Stay probability plots for *Q*(1) (**E**,**G**) and model-based (**F, H**) agents on the two task versions. Plots show the fraction of trials on which the agent repeated its choice following rewarded and non-rewarded trials with common and rare transitions (SEM error bars shown in red). (**I, J**) Performance (fraction of trials rewarded) achieved by *Q*(1) and model based agents, and by an agent which chooses randomly at the first step. Agent parameters in (**I**,**J**) have been optimised to maximise the fraction of rewarded trials.

Recent approaches to behavioural neuroscience derive substantial explanatory value from parametric variation of decision variables in the context of large decision datasets. It is therefore desirable to develop tasks which achieve these ends, but also exhibit the critical feature of outcome devaluation–namely the wherewithal to discriminate model-based and model-free RL. The two-step task [7] represents one recently popular approach to creating such a task, attracting a substantial number of human studies [8,11,12,31–43]. The task is so named because each trial consists of two distinct steps (see task diagram, Fig 1A). At the first step the subject chooses between two actions, termed action A and action B. After making this choice the subject reaches one of two second-step states termed state *a* and state *b*. Action A normally leads to state *a*, and action B normally leads to state *b*; however, on a randomly selected 30% of trials, a rare transition occurs, such that action A leads to state *b* and action B to state *a*. In each second-step state two further actions are available. The subject chooses one of these actions and either receives or not a reward before starting the next trial. The reward probabilities for each of the four second-step actions (two in each second-step state), vary over time as reflecting Gaussian random walks on the range 0.25–0.75 (Fig 1C).

Rewards obtained (or not) at the second step modify the subjects estimates of the values of the second-step states, which are themselves the outcomes of the first step actions. On trials with rare transitions, the second-step state whose value is changed by obtaining or not a reward is normally reached from the first-step action that was not chosen. This suggests that a model-based agent which understands the true mapping between first-step actions and second-step states will behave differently from a model-free agent which does not use this knowledge. Model-based and model-free control can indeed be dissociated by evaluating how the events on one trial, specifically the transition (common or rare) and outcome (rewarded or not), affect the probability of repeating the same choice at the first step on subsequent trials.

Three sorts of analysis of these effects are in common use. The simplest is to look directly at the probability of repeating the first-step choice on just the next trial–this is called the ‘stay’ probability. A model-free strategy in which the value of the chosen first step action is updated directly by the trial outcome produces a pattern in which the subject tends to stay following rewarded and switch following non-rewarded trials, with no effect of transition (Fig 1E). By contrast a model-based strategy in which the subject understands the transition structure linking the first step actions to second-step states produces a pattern of stay probabilities which show a transition x outcome interaction, i.e. rewards increase stay probability following common transitions and decrease stay probability following rare transitions (Fig 1F). A second, more sophisticated version of this analysis is to perform multiple logistic regression of the probability of choice on one trial based on facets of choice and outcomes on one or more previous trials. RL algorithms imply that events can have an impact multiple trials into the future; this analysis can test this. We will also see that using extra regressors can alleviate potential confounds in the differentiation of MB and MF strategies. Finally, a third analysis is to fit RL models to behaviour using likelihood-based methods, and to compare directly their quality of fit.

There is strong evidence that human subjects who have been explicitly told in advance about the transition structure and drifting reward probabilities, gamely pursue model based strategies, potentially integrating them with MF influences [7,8,34]. Given the unique set of attractive features offered by the two-step task, versions suitable for animal subjects would be desirable, and several groups are currently pursuing work in this direction (Miller at al. Soc. Neurosci. Abstracts 2013, 855.13, Groman et al. Soc. Neurosci. Abstracts 2014, 558.19, Miranda et al. Soc. Neurosci. Abstracts 2014 756.09, Akam et al. Cosyne Abstracts 2015, II-15). However, an informal observation, that we formalize below, is that the stochasticity of the conventional version of the task means that even optimised model-based strategies perform little better than chance level and do not outperform simple model-free strategies. Animal subjects are less tolerant when complex strategies have only limited advantages, and often switch to strategies such as always choosing the same option, or alternation, which obtain rewards at chance level with minimal cognitive effort. It will therefore likely prove necessary to increase the contrast between good and bad options in order to use the task with animal subjects, and our understanding is this is being done in the current crop of animal studies.

Here we consider a stripped down version of the task which substantially improves the payoff for model-based strategies relative to chance level and model-free control. We show that seemingly innocuous changes to the task induce correlations between events which can allow model-free RL to masquerade as model-based. We first show that correlation between action values at the start of trials and the subsequent trial events can cause the stay probability analysis, when applied to the behaviour of purely model-free agents, to exhibit the transition-outcome interaction classically interpreted as indicative of model-based RL. We further show that a previously proposed modification to the analysis [34] accurately corrects for these correlations.

A second, and more pernicious, issue arises from the correlation between where rewards are obtained (second-step state *a* or *b*), and the expected value of choosing action A or B at the first step. We explore the behaviour of two agents which exploit this correlation. The first uses the trial outcome and location on the previous trial as a discriminative stimulus for the state of the world, using model-free RL to learn separate values for actions A and B following each combination of outcome (rewarded or not) and second-step state (*a* or *b*) reached on the previous trial. The agent develops a fixed mapping from one trial’s events to the next trial’s choice (e.g. reward in state *a* ➔ choose action A), that generates behaviour that would be assessed as being model-based by either classical or improved stay probability analysis. The representation underlying the second agent makes explicit the latent or hidden state of the world–i.e. which second-step state has higher reward probability. The agent infers this hidden state by observing where it obtains rewards, and uses a fixed mapping from its estimate of the latent state to action. This agent produces behaviour which is qualitatively very similar to that of a model-based agent. Both agents outperform classical model-free strategies in terms of the fraction of rewarded trials; this provides an incentive for the acquisition of these alternative representations via the ample statistical evidence available particularly to over-trained animal subjects of the correlations that underpin them. These strategies can also in principle generate seemingly model-based behaviour on the original version of the task used in the human literature, and may play a role in the automatization of apparently model-based control recently reported to occur with extended training on the original task [42].

## Results

The original two-step task and a simplified version with enhanced contrast between good and bad options, termed the reduced two step task, are shown in Fig 1. There are three differences between the original and reduced task; firstly the probability of a common transition is increased from 0.7 to 0.8, secondly there is no choice at the second step but rather a single action available in each state, thirdly the reward probabilities in the two second-step states alternate between blocks with reward probabilities of 0.8/0.2 and blocks with reward probabilities of 0.2/0.8 in states *a*/*b* (Fig 1D).

We initially simulated the behaviour of a model-free and a model-based agent on both versions of the task. The model-free agent (strictly speaking a *Q*(1) agent, termed a Direct Reinforcement agent in [7]) updated the value of the chosen action (A or B) based on the prediction error between the trial outcome and its current estimate of the action value, using a fixed learning rate. This agent therefore only used information about whether or not the trial was rewarded, and did not use information either about whether a common or rare transition occurred or about the second-step state from which the outcome was received. By contrast, the model-based agent calculated the value of each action A or B as the weighted sum of the values of states *a* and *b*, where the weights were determined by the (known) conditional probabilities of reaching those states after choosing that action. In the original task, the value of the second-step states used by the model based agent was the higher of the two action values available in each state. In the reduced task the state value was the value of the single action available in that state. Both agents updated the value of the chosen second-step action based on the prediction error between the current estimate of its value and the trial outcome.

As reported previously for the original two-step task [7], the behaviour of *Q*(1) and model-based agents could be differentiated by looking at how the transition (common or rare) and outcome (rewarded or not) influenced the stay probability, which is the frequency with which the agent repeated the same action on the subsequent trial (Fig 1E and 1F). As the action value update used by the *Q*(1) agent is only sensitive to the outcome and not the transition, the stay probability was higher for rewarded than non-rewarded trials, but was not influenced by whether a common or rare transition occurred (Fig 1E). For the model-based agent (Fig 1F), a reward following a rare transition increases the value of the state that is more commonly reached from the action that was *not* chosen on that trial. This increases the probability that the agent switches its choice on the subsequent trial. Stay probabilities for the model-based agent therefore showed an interaction between outcome and transition, such that rewards increased stay probability (i.e., were reinforcing) after common transitions, but reduced stay probability after rare transitions.

We evaluated the performance, i.e. the fraction of rewarded trials, achieved by the model-based and *Q*(1) agents on the original version of the task when their parameter values were optimised (Fig 1I). The *Q*(1) agent obtained rewards on a fraction 0.558 ± 0.006 of trials while the model-based agent obtained reward on 0.557 ± 0.005 of trials (mean ± SEM). The difference in performance between the agents was not significant (P = 0.91) and the performance of neither agent was significantly different from that of an agent which chose randomly at the first step and used model-free RL at the second step (P > 0.52). Both agents (modestly) outperformed a purely stochastic agent that chooses randomly at both steps, and so receives reward on 0.5 of trials. These results show that the stochasticity of the original task imposes a low ceiling on achievable performance, preventing model-based control from outperforming simple model-free strategies. By contrast, on the reduced version of the task the *Q*(1) agent with optimised parameters obtained rewards on 0.594 ± 0.002 of trials while the model based agent obtained rewards on 0.649 ± 0.003 if trials (Fig 1J). This performance difference was significant (P < 10^−6^), confirming that the modifications made in the reduced task increased the contrast between good and bad options and differentiated the performance achieved by different strategies.

### Action values at trial start affect stay probabilities

**Fig 2:**
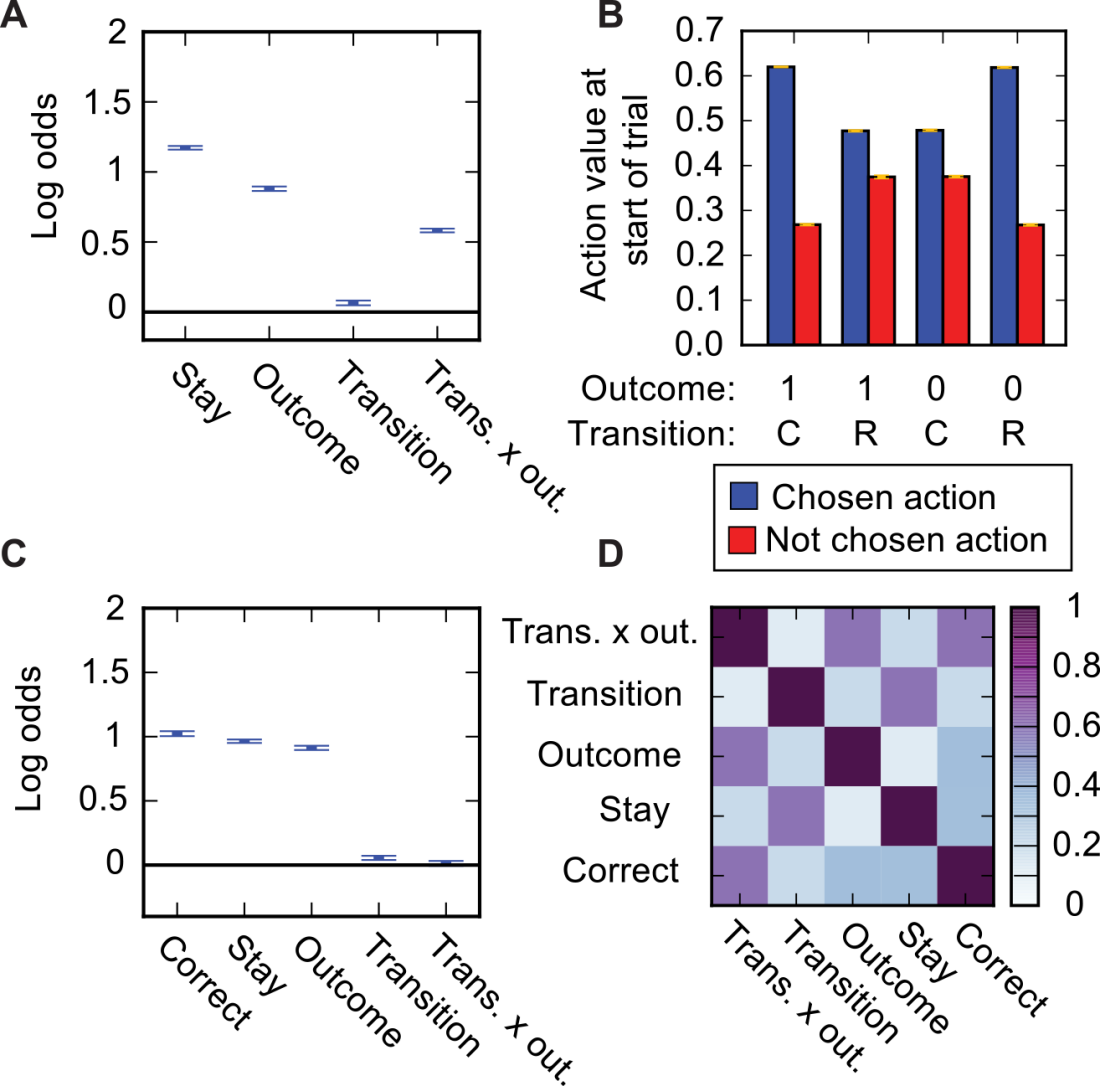
Stay probability transition-outcome interaction for *Q*(1) agent due to trial start action values. (**A**) Predictor loadings for logistic regression model predicting whether the *Q*(1) agent will repeat the same choice as a function of 4 predictors; Stay–a tendency to repeat the same choice irrespective of trial events, Outcome–a tendency to repeat the same choice following a rewarded trial, Transition—a tendency to repeat the same choice following common transitions, Transition x outcome interaction–a tendency to repeat the same choice dependent on the interaction between transition (common/rare) and outcome (rewarded/not). (**B**) Action values at the start of the trial for the chosen and not chosen action shown separately for trials with different transitions (common or rare) and outcomes (rewarded or not). Yellow error bars show SEM across sessions. (**C**) Predictor loadings for logistic regression model with additional predictor capturing tendency to repeat correct choices, i.e. choices whose common transition lead to the state which currently has high reward probability. (**D**) Across trial correlation between predictors in logistic regression analysis shown in (**C**).

**Fig 3:**
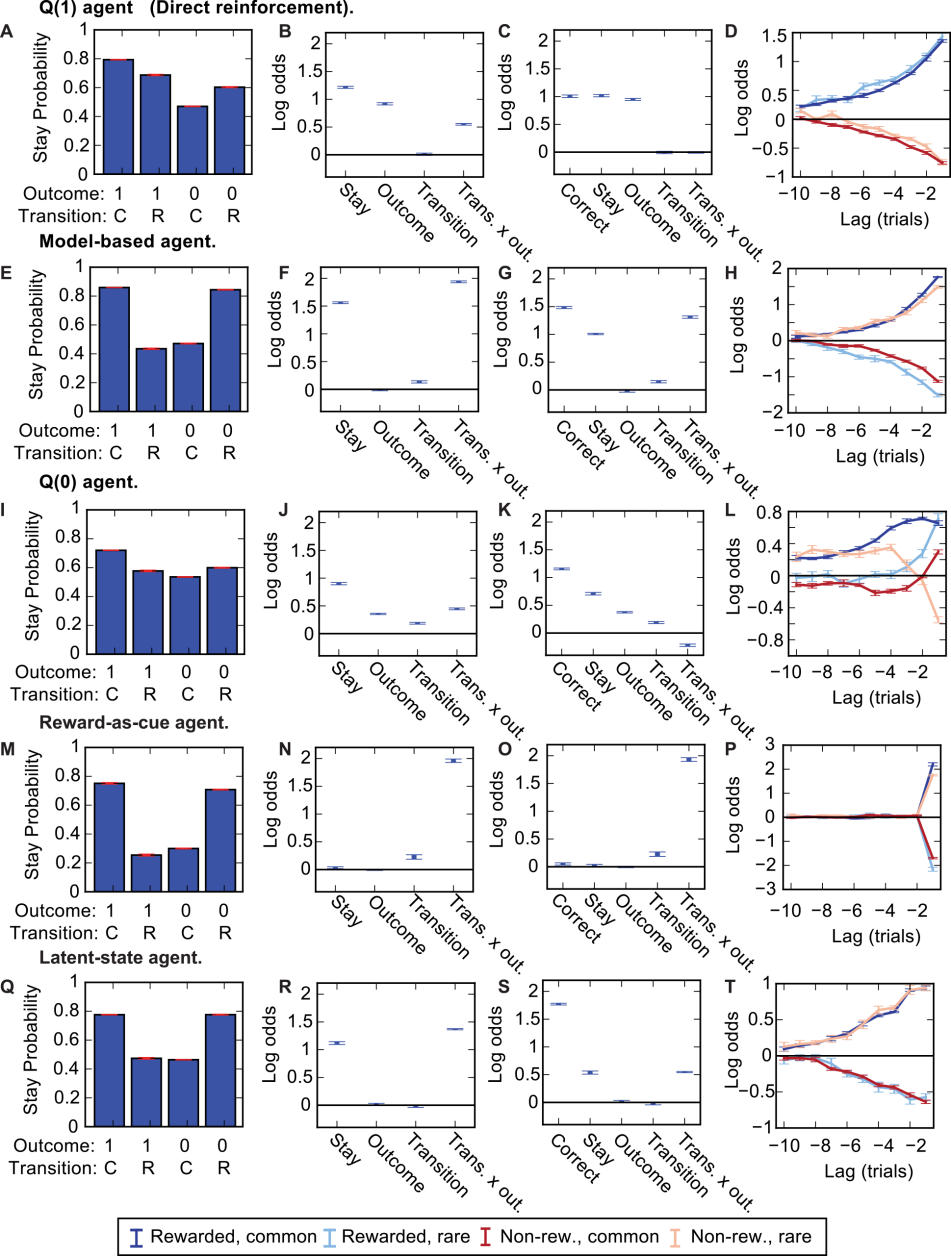
Comparison of agents’ behaviour–reduced task. Comparison of the behaviour of all agents types discussed in the paper on the reduced task. Far left panels–Stay probability plots. Centre left panels—Predictor loadings for logistic regression model predicting whether the agent will repeat the same choice as a function of 4 predictors; Stay–a tendency to repeat the same choice irrespective of trial events, Outcome–a tendency to repeat the same choice following a rewarded trial, Transition—a tendency to repeat the same choice following common transitions, Transition x outcome interaction–a tendency to repeat the same choice dependent on the interaction between transition (common/rare) and outcome (rewarded/not). Centre right panels–Predictor loadings for logistic regression analysis with additional ‘correct’ predictor which captures a tendency to repeat correct choices. Right panels—Predictor loadings for lagged logistic regression model. The model uses a set of 4 predictors at each lag, each of which captures how a given combination of transition (common/rare) and outcome (rewarded/not) predicts whether the agent will repeat the choice a given number of trials in the future, e.g, the ‘rewarded, rare’ predictor at lag -2 captures the extent to which receiving a reward following a rare transition predicts that the agent will choose the same action two trials later. Legend for right panels is at bottom of figure. Error bars in all plots show SEM across sessions. Agent types: (**A-D**) *Q*(1), (**E-H**) Model-based, (**I-L**) *Q*(0), (**M-P**) Reward-as-cue, (**Q-T**) Latent-state.

Behaviour simulated from the *Q*(1) agent on the reduced version of the task showed a strikingly different pattern of stay probabilities from that seen in the original task (Fig 2G, repeated for convenient comparison in Fig 3A). Stay probabilities showed a clear interaction between transition and outcome. A logistic regression analysis predicting stay probability as a function of outcome, transition, and transition-outcome interaction confirmed that transition-outcome interaction predicted stay probability (P < 10^−10^, t-test for non-zero predictor loading) (Fig 2A), and this predictive relationship held true over a wide range of agent parameter values (S1A Fig). This result is counter-intuitive because by construction, the action values and hence choice probabilities of the *Q*(1) agent are unaffected by whether a common or rare transition occurred. The difference in stay probability between trials with the same outcome but different transitions therefore cannot be accounted for by a difference in the action value update that occurred on that trial, as the update is identical irrespective of the transition. Instead, the reason why the action values of the chosen and non-chosen option are (on average) different following trials with the same outcome but different transitions must be that the action values at the start of the trial are (on average) different. This can indeed be seen (Fig 2B); the mean difference between the action values for the chosen and not chosen option at the start of the trial was larger for common-rewarded and rare-not rewarded trials than for common-not rewarded and rare-rewarded trials.

Why are action values at the start of the trial correlated with subsequent trial events, specifically the transition-outcome interaction? There are two steps in the argument. First, the difference in action values between chosen and not-chosen action is on average larger for trials where the agent chooses the correct action, i.e. that which commonly leads to the state with high reward probability, than for trials where the agent choses the incorrect option. When the difference in action values is small, the agent has little evidence that one option is better than the other, and is more likely to choose the incorrect action. Additionally, due to the stochastic softmax decision rule the agent sometimes chooses the action with lower subjective value, and such ‘exploratory’ choices are more likely to be incorrect. Second, choosing the correct, rather than incorrect, action changes the probabilities of observing different combinations of trial events. Rewarded common transitions and unrewarded rare transitions are more likely to occur following a correct action than they are to occur following an incorrect action. Conversely, rewarded rare transitions and unrewarded common transitions are more likely to occur following an incorrect action.

To summarise; the difference in action values going into the trial correlates with the probability of choosing the correct option. Whether the agent chooses the correct option determines the probability of observing each combination of subsequent trial events. Therefore when trials are divided into groups by outcome and transition, the action values at the start of the trial show a transition-outcome interaction (Fig 2B), which is then also observed for the stay probabilities (Fig 1G), even though the agent did not use any information about the transition in its action value update.

This effect is not restricted to block based reward probabilities; it can also be observed when reward probabilities change as random walks (S2A and S2B Fig), or with static fixed reward probabilities of 0.8 / 0.2 in states *a* / *b* (S2F and S2G Fig). When reward probabilities in the two second-step states are fixed and equal (S2H and S2I Fig), the *Q*(1) agent shows no transition-outcome interaction as action values at the start of the trial differ only through stochastic fluctuations in the experienced outcomes and are therefore not correlated with subsequent trial events.

Data simulated from the *Q*(1) agent on the original version of the task does in fact show a significant (P = 0.01) albeit very small positive loading on the transition-outcome interaction predictor (S3B Fig) due to the mechanism outlined above. The effect is radically weaker than in the reduced task because the greater stochasticity in the state transitions and reward delivery in the original task greatly reduce the strength of correlation between action values at the first step and subsequent trial events. As this effect is so weak in the original task we do not consider it to have any implication for the stay probability analyses in the existing human literature.

### Correction to stay probability analysis

It is possible to modify the logistic regression analysis of stay probabilities to prevent differences in action values at the start of the trial from appearing as a spurious loading on the transition-outcome interaction predictor. This can be done by including an additional ‘correct’ predictor which captures the tendency of the agent to repeat correct choices, as originally suggested in [34]. Including this additional predictor completely removed loading on the transition-outcome interaction predictor for the *Q*(1) agent (P = 0.67, t-test for non-zero predictor loading) (Fig 2C; repeated for convenient comparison in Fig 3C), correctly revealing that only the trial outcome affected the agent’s subsequent choice. For the model-based agent this extended logistic regression analysis showed positive loading on the transition-outcome interaction predictor (P < 10^−12^) (Fig 3G) reflecting the true importance of this interaction to the action value update used by the agent. Including the correct predictor did reduce loading on the interaction predictor for the model-based agent by 32.3%, indicating that trial start action values also contributed to the pattern of stay probabilities for this agent. The addition of a correct predictor works because the correlation between actions values at the start of a trial and the subsequent transition-outcome interaction is entirely mediated by the correlation between these action values and whether the agent chose the correct action on that trial. Explicitly including a predictor for repeating correct choices absorbs the variance due to action values at the trial start which would otherwise be absorbed by the transition-outcome interaction predictor due to correlation between these two predictors (Fig 2D).

Including the correct predictor reduced, but failed to completely remove, loading on the transition-outcome interaction predictor for the *Q*(1) agent simulated on the reduced task version with random walk reward probabilities (P < 10^−3^, t-test for non-zero predictor loading) (S2D Fig). We hypothesised that the correct predictor failed to correctly compensate for trial start action values because it did not reflect the magnitude of the difference in reward probabilities between the two second-step states. We therefore tried using a continuous valued correct predictor whose magnitude was given by this difference. Including this predictor completely removed loading from the transition-outcome interaction predictor (P = 0.78, t-test for non-zero predictor loading) (S2E Fig).

An alternative way of differentiating model-based and model-free strategies is a lagged logistic regression analysis which examines the effect on choice probability of trial events at different lags relative to the current trial (Miller at al. Soc. Neurosci. Abstracts 2013, 855.13). Fig 3D and 3H show a lagged logistic regression analysis for *Q*(1) and model-based agents. The analysis evaluated how different combinations of outcome and transition predict that the agent will repeat the same choice a given number of trials in the future. For example, the ‘rewarded, rare’ predictor at lag -2 captures the extent to which receiving a reward following a rare transition predicted that the agent will choose the same action two trials later. This analysis is therefore an extension of the classical stay probability analysis to include the effect of earlier trials. For the *Q*(1) agent (Fig 3D), obtaining a reward predicted that the agent will repeat the same choice irrespective of the transition, with a smoothly decreasing predictive weight at increasing lag. For the model-based agent (Fig 3H), rewarded-common transitions and non-rewarded rare transitions predicted the agent will repeat the same choice, while rewarded-rare and non-rewarded common transitions predict the agent will not repeat the same choice, again with the predictive weight smoothly decreasing with increasing lag.

### Model-based and model free agent variants

Various other factors have been suggested as influencing strategies, including eligibility traces for MF algorithms, the possibilities of continual learning of the transition probabilities, and also outcome- and transition-independent perseveration. We also considered the effects of all of these on the statistics of choice.

Although a *Q*(1) agent is typically used to illustrate model-free behaviour on the two-step task, it represents one end of a spectrum of model-free agents differentiated by the extent to which the action value update at the first step depends on either the trial outcome or second-step action values. This spectrum is parameterized by the eligibility trace parameter conventionally called *λ*. The update used by the *Q*(1) agent depends only on the trial outcome and not at all on the values of the second-step state (or second-step actions in the original two-step task [7]). At the other end of the spectrum is the *Q*(0) agent which updates the value of the first step action based only on the value of the second-step state, with no direct influence of the trial outcome. The value of the second-step state is then updated based on the trial outcome. The behaviour of a *Q*(0) agent on the simplified two-step task is shown in Fig 3I–3L, and on the original task in S3I–S3L Fig. The behaviour on the reduced task of model-free agents which use mixtures of the *Q*(1) and *Q*(0) updates are shown in S4 Fig. The one trial back extended logistic regression analysis for the *Q*(0) agent (Fig 3K) shows positive loading on the transition and outcome predictors and negative loading on the transition-outcome interaction predictor. Loading on the transition-outcome interaction predictor in the extended logistic regression analysis distinguishes the model-based agent from model-free agents across the range of values of *λ*, none of which shows positive loading on this predictor. The lagged logistic regression for the *Q*(0) agent shows a complex pattern in which the predictive weight of each combination of trial events does not decay smoothly at increasing lags.

It is typically assumed that subjects on the two-step task understand that the transition probabilities linking the first step actions to second-step states are fixed, and hence do not update their estimates of these based on the transitions they experience trial to trial. As this assumption may not be valid for subjects who do not have prior information about the task structure, we evaluated the behaviour of a model-based agent which learned the transition matrix online by updating its estimate of the transition probabilities for the chosen action on each trial based on the experienced transition (S5 Fig). With a low transition learning rate, such that the estimates of the transition probabilities averaged over many prior trials, the behaviour of the agent was similar to that of the model-based agent with fixed transition probabilities (S5A–S5D Fig). At higher transition learning rates, loading on the transition-outcome interaction predictor decreased, while loading on the outcome and transition predictors increased (S5G Fig). At high transition learning rates where the agent’s estimate of the transition probabilities was dominated by the most recently experienced transition, loading on both the outcome and transition predictors was substantially higher than that on the transition-outcome interaction predictor (S5L Fig).

Human subjects typically show a perseveration bias on the two-step task [7,8], i.e. a tendency to repeat first step choices independent of the trial events. We therefore tested how a perseveration bias affected behaviour on the reduced task for Q(1) and model-based agents (S6 Fig). Perseveration bias increased stay probability (S6A and S6E Fig) and loading on the stay predictor (S6C and S6G Fig), but did not change the characteristic pattern of positive loading on the outcome predictor for the Q(1) agent and the transition-outcome interaction predictor for the model-based agent (S6C and S6G Fig).

### Extended state representations

We have so far considered only agents whose state representation corresponds to that used by the experimenter to define the task. However, identifying those states that are relevant for behaviour is a substantial component of the real control problem faced by organisms and there is no guarantee that when faced with a decision task, subjects will adopt the same state representation conceived by the experimenter. In the two-step task there is an underlying latent state that is relevant to behaviour–whether the reward probabilities are higher in state *a* or *b*. This induces correlation between where rewards are obtained and the true expected value of first step actions. It turns out that model-free agents that exploit these correlations or even attempt to learn this full latent structure, can produce behaviour similar to that of a model-based agent without using the prospective action evaluation that is the hallmark of classical model-based RL.

We first consider a simple way of exploiting the correlations. The two-step task has a circular structure in which subjects cycle repeatedly through the decision state, second-step states and trial outcomes. This repeating structure provides opportunities for subjects to learn predictive relationships between events on one trial and the actions that are likely to lead to reward on the subsequent trial. One such predictive relationship is that the location where reward is obtained on one trial predicts which choice on the next trial is likely to lead to reward. That is, if a reward is obtained in state *a*, the reward probability is higher for choosing action A on the subsequent trial, while if reward is obtained in state *b* the reward probability is higher for choosing action B on the subsequent trial. Note that this predictive relationship holds true across reversals in which second-step state has higher reward probability. The locations where reward is obtained, and conversely where non-rewards are obtained, can therefore, in principle, be used as discriminative stimuli to guide choice on the next trial. We therefore considered the behaviour of a ‘reward-as-cue’ agent which uses the location of reward as a discriminative stimulus for the state of the world. Specifically, the reward-as-cue agent treated the choice between actions A and B as occurring in one of 4 distinct states on each trial, defined by whether a reward or non-reward was obtained in state *a* or *b* at the end of the previous trial. The agent used model-free RL to learn independent values of actions A and B in each of these 4 states. Like the *Q*(1) agent, the reward-as-cue agent updated the value of the chosen action dependent on reward prediction error between its current estimate of the action value and the trial outcome, without using the action value at the second step in the update. The agent learned action values which produced the strategy of choosing action A following rewards in state *a*, action B following rewards in state *b*, action B following no reward in state *a* and action A following no reward in state *b*. This corresponds to a strong stay probability transition-outcome interaction (Fig 3M–3O). Unlike the other agents considered so far, the reward-as-cue agent does not adapt to changes in the reward probabilities across blocks through changes in its action values. Rather, the action values are stable across blocks and reflect a fixed mapping between where reward is obtained and which action should be taken on the next trial.

It is plausible that over-trained animals could learn to use the location of reward as a discriminative stimulus to guide choice on the next trial, as animals straightforwardly learn to use discriminative sensory stimuli of various sorts as cues for the best action to take next [44–47]. Once learnt, this strategy would be minimally cognitively demanding as it is essentially a fixed stimulus-response habit with only a limited demand on working memory. However, although the reward-as-cue agent gives behaviour on the one trial back stay probability analysis which is qualitatively similar to that of a model-based agent, it shows a very different pattern of loadings in the lagged logistic regression analysis (Fig 3P). Rather than the smooth drop off of predictive weight with increasing lag observed for the model-based agent, only the previous trial events are predictive of the reward-as-cue agent’s behaviour.

**Fig 4:**
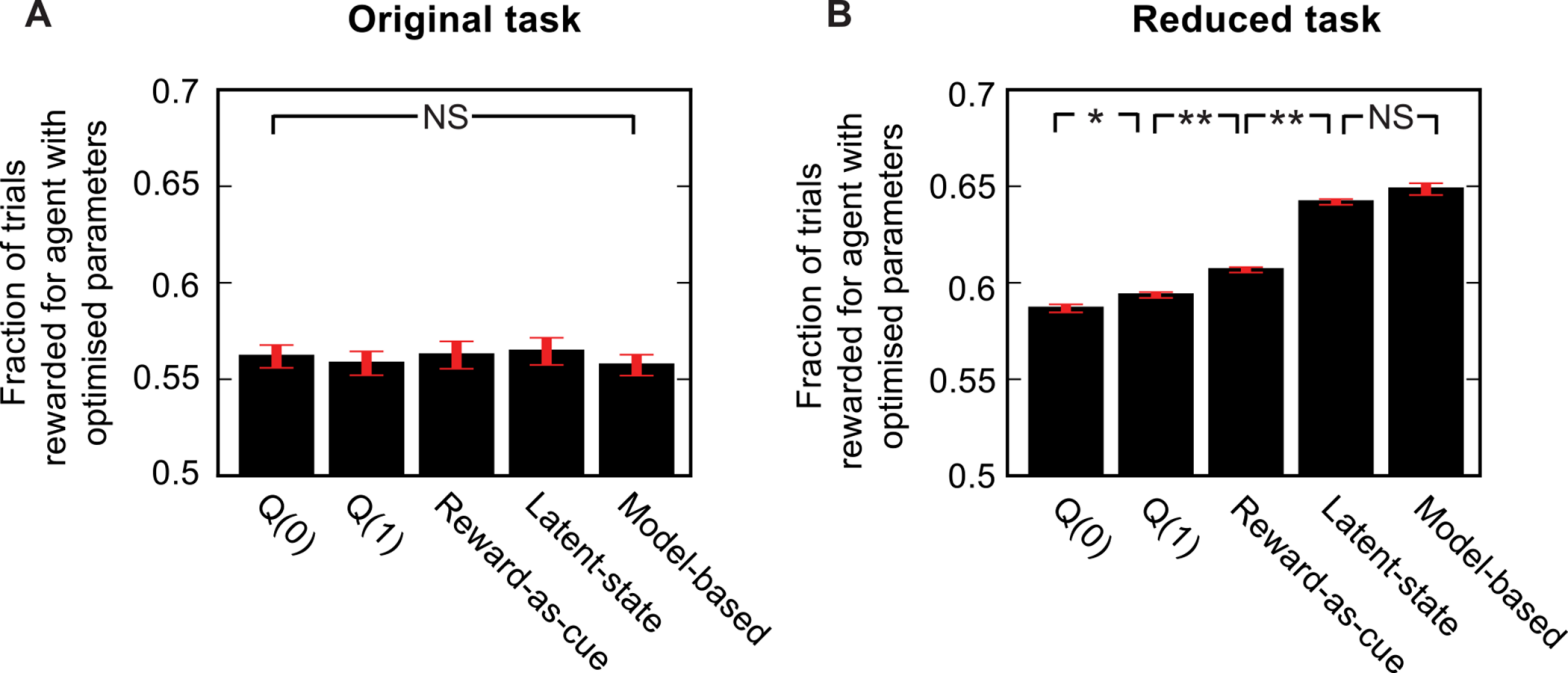
Comparison of agents’ performance. Performance achieved by different agent types in the original (**A**) and reduced (**B**) tasks, with parameter values optimised to maximise the fraction of trials rewarded. For the reward as cue agent, performance is shown for a fixed strategy of choosing action A (B) following reward in state *a* (*b*) and action B (A) following non-reward in state *a* (*b*). SEM error bars shown in red. Significant differences indicated by *: 5 < 0.05, ** P < 10^−5^.

For all the agents in Fig 3, we chose parameters determined by a maximum likelihood fit to the behaviour of the model-based agent, so that they would all have comparable average behaviour; see materials and methods). For the reward-as-cue agent, this suggested a very low learning rate (0.003). If, instead, we chose parameters for all agents that maximized the fraction of trials that are rewarded, the reward-as-cue agent outperformed both *Q*(1) and *Q*(0) model-free agents (Fig 4B).

As noted, the reward-as-cue strategy works because there is in fact a latent, unobservable state of the world that is important to the decision problem–whether the reward probability is higher in state *a* or *b*. The location where reward is obtained is correlated with, and hence informative about, this latent state, and therefore can be utilised as a discriminative stimulus to guide behaviour. However, because the reward-as-cue strategy uses only the most recent reward as a discriminative stimulus, it is far from optimal. We therefore evaluated the behaviour of a different agent we term ‘latent-state’ which understands that the world is always in one of two latent states, one in which the reward probability is high in state *a* and low in state *b*, and the other in which the reward probability is high in state *b* and low in state *a*. At the end of each trial the latent-state agent performed a Bayesian update of its estimate of the probabilities that world is in each latent state based on the observed trial events. In updating the probabilities the agent also assumed that there is a small probability (the inverse of the mean block length) that the state of the world switches between trials. This amounts to the assumption that the block lengths are exponentially distributed, rather than being of fixed length, as generally employed. We did not explicitly model the learning of action values in each of these latent states, but rather assumed asymptotic behaviour in which the agent chose action A with high probability in the latent state where state *a* had high reward probability and action B with high probability in the latent state where state *b* had high reward probability.

**Fig 5:**
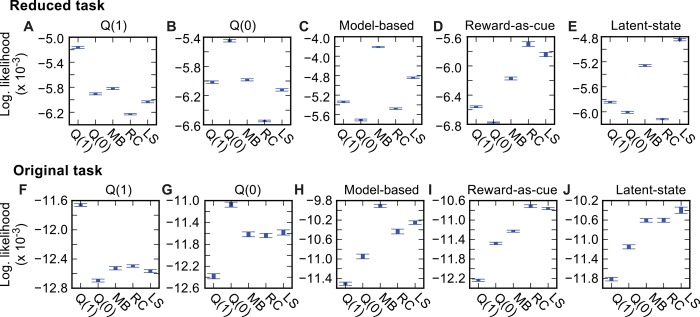
Likelihood comparison. Data likelihood for maximum likelihood fits of different agent types (indicated by x-axis labels; MB–Model based, RC–Reward-as-cue, LS–Latent-state) to data simulated from each agent type (indicted by labels above axes) on the reduced (**A-E**) and original (**F-J**) tasks. All differences in data likelihood between different agents fit to the same data are significant at P < 10^−4^ except for that between the fit of the reward-as-cue and latent-state agents to data simulated from the reward-as-cue agent which is significant at P = 0.027.

The behaviour of the latent-state agent looked qualitatively very similar to that of the model-based agent. The one trial back stay probability analyses showed a transition-outcome interaction (Fig 3Q–3T). As for the model-based agent (Fig 3H), the lagged logistic regression analysis for the latent-state agent (Fig 3T) showed a tendency to repeat choices that were followed by rewarded common and non-rewarded rare transitions, and to not repeat choices that were followed by non-rewarded common and rewarded rare transitions, with a gradually decreasing predictive weight at increasing lag. However, the behaviour of the latent-state and model-based agents could be discriminated using model fitting, with data simulated by the model-based agent being fit with higher likelihood by the model-based agent (Fig 5C) and data simulated by the latent-state agent being fit with higher likelihood by the latent-state agent (Fig 5E). Data simulated from either latent-state or model-based agents was better fit by both of these agents than by either the *Q*(0) or *Q*(1) model-free agents. All agents on the reduced task had the same number of parameters, so model comparison using data likelihood directly, without correction for model complexity, is appropriate.

With parameters optimized to maximize reward, the latent-state agent achieved performance that was not significantly different from the model-based agent (Fig 5B).

Finally, we evaluated the behaviour of agents using the reward-as-cue and latent-state strategies on the original version of the task (S3M–S3T Fig). The reward-as-cue strategy produced a weak but significant (P = 0.01) transition-outcome interaction effect on stay probability (S3M and S3O Fig). The transition-outcome interaction is much weaker than that observed for the reduced task because the location where rewards are obtained is more weakly correlated with the expected value of the first step actions–obtaining a reward in state *a* is in fact only weakly correlated with a high expected value of action A. For the latent-state strategy we assumed the agent believed that at any point in time, reward probability in one second-step state is 0.625 (the 75th percentile of the range of reward probabilities) and in the other 0.375 (the 25th percentile). This agent produced behaviour that was qualitatively very similar to the model-based agent (S3Q–S3T Fig). No significant difference was observed in the performance (fraction of trials rewarded) achievable by any of the different strategies considered on the original task version (Fig 5A). As in the reduced task, data simulated from each agent was fit with higher likelihood (Fig 5F–5J), and lower BIC score (S7 Fig), by that agent than by any of the other agents.

## Discussion

We have provided a detailed analysis of the performance of a number of different RL strategies on variants of the two-step task. Since in the original task, complex and taxing strategies only garner modestly more reward than simple ones, it might seem attractive to alter the task to enhance the discrimination. We showed some dangers inherent in this idea, in that induced correlations can make discrimination harder. We also generalized this analysis to more complicated model-free strategies.

In particular, we identified two ways in which behaviour on the two-step task could, under certain conditions, be incorrectly identified as arising from prospective model-based evaluation of actions. The first issue is with the stay probability analysis commonly used as a metric of subjects’ strategies. We showed that rather than reflecting only the action value update occurring on a given trial, which is distinct for model-based and model-free action evaluation, stay probabilities can also be affected by action values at the start of the trial. This can cause the behaviour of a model-free agent to exhibit a stay probability transition-outcome interaction, which is classically interpreted as a signature of model-based behaviour. The second issue is the existence of alternative strategies which use different state representations from the basic states that define the task structure and produce behaviour which is similar to that of a model-based agent though not dependent on prospective evaluation of the outcome of actions.

The possibility that purely model-free agents can exhibit a transition-outcome interaction effect on stay probability has been discussed in two prior studies using the original two-step task [12,34]. Both studies suggested this can occur when the reward probabilities at the second step change slowly, with [12] further stating that a large initial difference in reward probabilities between the two second-step states contributes to the effect. Our analysis clarifies the mechanism by demonstrating that it is due to correlation between action values at start of the trial, specifically the difference in action values between the chosen and not chosen action, and subsequent trial events. The effect is very weak in the original task where both the state transitions and reward delivery are highly stochastic, and hence decorrelate action values and subsequent trial events. Our results show the effect can be strong even when reward probabilities change rapidly, as the reduced task version with random walks showed a strong effect (S2A–S2E Fig) even though the standard deviation of random walk step sizes was 4 times larger than in the original task.

The effect of trial start action values can be corrected for in a logistic regression analysis of stay probabilities by including a predictor which captures the tendency to choose the action which was correct on the previous trial, i.e. to repeat correct choices. Such a modification to the regression analysis was proposed in [34] and our results demonstrate its efficacy. We suggest this modified stay probability analysis will be a useful tool for evaluating the influence of trial events on subsequent choice in task variants where the classical stay probability analysis gives misleading results. One cost of using this additional predictor is that, as it is correlated with the transition-outcome predictor (Fig 2E), the relative loadings on these two predictors will be sensitive to fluctuations in the data, potentially requiring larger datasets to achieve reliable results.

The second issue we have identified with the two-step task is that, due to its repeating structure, subjects could, in principle, learn to exploit correlations between where rewards are obtained and the expected value of first step actions, to produce behavioural strategies that look similar to model-based behaviour but do not use prospective evaluation of actions. One simple strategy which we termed ‘reward-as-cue’ learns a fixed mapping between events on one trial and choice on the next trial (e.g. reward in state *a* → choose action A). Notably, it outperformed classical model-free strategies in terms of acquiring reward. The attraction of this strategy from the point of view of the subject is that once learned it requires no further updating of action values to adjust to changes in reward probability in the two second-step states, and hence can be fully automatized into a stimulus-response habit. Though this strategy produces a strong stay probability transition-outcome interaction, it can be distinguished from model-based behaviour as only the most recent trial influences choice. However behaviour strikingly similar to that of a model-based agent was generated by a more sophisticated strategy we termed ‘latent-state’, which uses the location of recent rewards as a discriminative stimulus for which of two latent states the world is in (high reward probability in state *a*, or high reward probability in state *b*), and follows a fixed mapping from the latent state of the world to choice.

On the large simulated datasets used in this study, behaviour simulated from latent-state and model-based agents could be differentiated by model-comparison, and this probably represents the best approach to doing so in experimental data. Data simulated from the latent-state agent was fit with higher likelihood (Fig 5E and 5J) and lower BIC score (S7E Fig) by this agent than by the model-based agent, and vice versa. Though the differences were small, particularly on the original task, they were highly significant (P < 10^−5^). However, several factors will make this discrimination more difficult when working with experimental data. Firstly; the size of experimental datasets is typically substantially smaller, reducing the resolution of model comparison approaches. Secondly, the quantitative details of fitted models are unlikely to exactly match subject’s strategies. Thirdly, subject’s behaviour may be generated by a mixture of interacting control systems using different strategies. Whether latent-state and model-based strategies can be discriminated using model comparison in a given behavioural dataset is ultimately an empirical question.

Is it plausible that subjects could learn latent-state type strategies in the two-step task? Many paradigms for humans and animals show evidence of aspects of this. It is apparent in probabilistic reversal learning tasks, in which humans [48] and monkeys [49] learn that there are in fact two distinct latent states of the world and use inference about the current latent state to guide their behaviour. Further, the huge wealth of tasks involving integration of noisy sensory evidence such as random dot motion discrimination and the Poisson clicks auditory discrimination task [44,46]. Take the former. Here, the latent state concerns which of two directions of motion is more prevalent in the input. Noisy sensory evidence is accumulated to draw this conclusion. In our task, the latent state is which of two states (*a* or *b*) is associated with a higher prevalence of reward. Noisy evidence, in the form of actual rewards, can be accumulated to draw an equivalent conclusion. Certainly there are important differences between these tasks; the timescale of integration is longer in the two-step and spans multiple trials, the discriminative stimuli in the two-step are themselves rewards, and the subjects take an active role in sampling the two information streams. However the inferential commonality is striking.

Both the reward-as-cue and latent-state strategies (termed collectively ‘extended-state’ strategies) work by exploiting the regularity in the task structure that the location where rewards are obtained correlates with which first step action has higher reward probability. Evidence for this regularity accrues slowly as it is only across multiple reversals in the reward probabilities that the correlation becomes apparent. It therefore seems probable that if subjects do learn to exploit this regularity, the strategy would only arise after extended experience with the task. In the original version of the task used typically in the human literature, subjects do a total of ~200 trials. The limited number of trials performed, and the fact that human subjects have been trained to understand the true task structure—presumably priming the use of a model-based strategy—both argue against the possibility that the apparently model-based behaviour reported in the bulk of the human literature in fact arises from extended-state strategies. Indeed, it is only after substantial additional training [42] that apparently model-based human two-step behaviour becomes resistant to inference from cognitive load from a demanding secondary task performed in parallel [11]. This training might lead to the creation of an extended-state strategy in which prospective model-based evaluation is replaced by a process of latent state inference with static state-action mappings, and thereby apparent automatization.

Latent state strategies go beyond classical model-free RL and are interesting in their own right. Indeed, although they do not use a model which predicts future state given chosen action, which following [3] we take as the definition of model-based RL, the latent state representation is a form of world model which allows the agent to approximate the behaviour generated by planning without the computational costs of simulating behavioural trajectories. In this respect it is similar to the successor representation [50], which generalises between actions based on the similarity of their experienced successor states, and can also approximate planning at reduced computational cost. Strategies like this illustrate the observation that the distinction between model-based and model-free RL is perhaps better thought of as a spectrum than a binary classification [51]. Nonetheless, the distinction between strategies that do and do not utilise a prospective model for predicting the future state given the chosen action is of interest, and we therefore suggest that in the design and interpretation of versions of tasks like the two-step, the possibility subjects may utilise extended-state strategies should be considered. This is of particular importance for versions intended for animal subjects, since the extensive training that typically precedes recordings or manipulations provides ample opportunity for task regularities to be learnt. Further, adaptations of the task to create sufficient contrast between good and bad options to offer sufficient incentive can provide stronger statistical evidence for the regularity that underpins extended-state strategies.

Various options exist to minimise the probability that apparently model-based behaviour is in fact due to such strategies. One would be to avoid overtraining subjects, limiting the total number of trials they perform. However, this precludes generating very large behavioural datasets to better quantify the effect of manipulations or the relationship between behaviour and neural activity. A second possibility is to accept that it may be difficult to disambiguate extended-state from classical model-based strategies purely from behaviour, and use neural data to try and disambiguate the strategy used by subjects. A final potential option is to modify the two-step task to introduce reversals into the transition matrix which maps the first step choice to second-step state. In this task variant, not only does the reward probability in each second-step state change over time, but the action which must be chosen to reach a given second-step state also changes. Model-based control that performs incremental learning of the current transition probabilities (one of the variants discussed above), can adjust in a straightforward manner to this change; one could even imagine coupling simple latent state inference for just the transition structure (as in conventional probabilistic reversal learning) to model-based RL. However, the task modification substantially increases the complexity of pure latent state strategies. Reversals in the transition matrix break the fixed predictive relationship in the original task between where reward is obtained and which action at the first step is likely to lead to reward. To solve this version through a fixed mapping from an inferred latent state to action requires latent states that are non-linear combinations of where rewards have been obtained and which actions have led to which states.

The possibility we have identified here for model-free strategies to masquerade as model-based mirrors proposals that apparently model-free behaviour on the two-step task may in fact be due to model-based selection applied to action sequences [12]. Though very different in their underlying mechanisms, both indicate the complexity of cleanly dissociating the contribution of different learning strategies to behaviour.

The two-step task latent state strategy provides an example of how agents may turn a planning problem into a set of automatized state-response mappings if there is a limited set of relevant states of the world, each with their own appropriate response. Even if the planning problem is large, with a great diversity of possible solutions, e.g. navigating from home to work, with experience the decision may be automatized to a mapping from a small number of relevant states of the world, e.g. is it rush hour, to a set of options which are known to work best in each condition. Such automatization is more sophisticated than stimulus-response habits as typically envisioned; the states of the world that evoke the response may be high level abstractions rather than directly observable stimuli, and the responses may be action sequences, or options in the hierarchical RL formalism [52]. However, as cached state–action mappings learnt through a history of reinforcement, such strategies have commonalities with classical habits and may be learnt using similar model-free RL algorithms applied to higher level state and action representations, perhaps instantiated in cortical-basal ganglia loops involving higher level cortices and associative and limbic striatal sub-regions. These considerations bring to the forefront the question of what state representations are learned and used [53,54], something known to be central to the speed with which agents learn to solve decision problems.

## Materials and Methods

### Simulations

All simulations and analysis were conducted in Python. Full code used to produce the paper figures is included in supplementary material (S1 Code). For each agent, 10 sessions of length 10000 trials were simulated. All trials were included in analyses. Where errorbars are used these show standard error of the mean across session.

### Tasks

All tasks used in the paper shared the common structure that on each trial an initial choice between two actions, termed action A and action B, led probabilistically to one of two states, termed state *a* and state *b* (see diagrams; Fig 1A and 1B). Action A normally lead to state *a* and action *B* normally lead to state *b*, but with fixed probability on each trial, a rare transition could occur such that action A lead to state *b* and action B to state *a*.

The following variants of the two-step task were used in the simulations:

#### Original task

Version of the task described in [7] and used in the majority of human studies. The probability of common/rare transitions was 0.7/03. Two actions were available in each second-step state and the subject chose one of these on each trial. The reward probabilities for the 4 second-step actions changed over time as a reflecting Gaussian random walk on the range 0.25–0.75, with the standard deviation of step size on each trial set to 0.025 (Fig 1C).

#### Reduced task

The probability of common/rare transitions was 0.8/0.2. There was one action available in each second-step state. Except where stated otherwise, reward probabilities alternated every 50 trials between blocks with reward probability 0.8/0.2 in states *a*/*b* and bocks with reward probability 0.2/0.8 in states *a*/*b*. S2 Fig used variants of the reduced task with different reward probability distributions in the second steps. In S2A–S2E Fig reward probabilities varied as Gaussian random walks on the range 0–1 with step size standard deviation of 0.1. In Fig 2F and 2G reward probabilities in states *a* and *b* were fixed at 0.2 and 0.8. In Fig 2H and 2I reward probabilities in states *a* and *b* were fixed at 0.5 and 0.5.

### Agents

In describing the action value updates used by the different agents we use the following variables:

*Q*(*s*_1_, *a*_1_): The value of the first step action chosen on the trial.

*Q*(*s*_2_, *a*_2_): The value of the second-step action chosen on the trial.

*r*: The trial outcome (1 for reward, 0 for non-reward).

*α*: The agent’s learning rate.

All agents used a softmax decision rule with inverse temperature parameter *T* to determine choice probabilities as a function of action values except for the first step choice of the latent state agent.

The update rules used by the agents were as follows:

#### *Q*(*λ*) agents

The action value update rules used by the *Q*(*λ*) agents (including *Q*(0) and *Q*(1) agents) were:

$$Q(s_{1},a_{1})\leftarrow \;(1-\alpha )Q(s_{1},a_{1})+\;\alpha \;\left(Q(s_{2},a_{2})+\;\lambda \;(r-Q(s_{2},a_{2}))\right)$$

$$Q(s_{2},a_{2})\leftarrow \;(1-\alpha )Q(s_{2},a_{2})+\;\alpha \;r$$

Where *λ* is the eligibility trace parameter (0 for the *Q*(0) agent and 1 for the *Q*(1) agent). Action value updates for *Q*(*s*_1_, *a*_1_) and *Q*(*s*_2_, *a*_2_) were applied sequentially at the end of each trial.

#### Model-based agent

At the start of each trial the model-based agent computed action values for the first step actions as:

$$Q(s_{1},a_{i})=\sum_{j}\;P\left(s_{j}|a_{i}\right)V\left(s_{j}\right)$$

Where *Q*(*s*_1_, *a*_*i*_) is the value of first-step action *i*, *V*(*s*_*j*_) is the value of the second-step state *j*, and *P*(*s*_*j*_|*a*_*i*_) is the true probability of reaching second-step state *j* after choosing action *i*. In the original task with a choice between two actions at the second step, the second-step state value *V*(*s*_*j*_) was the maximum of the two action values available in that state; *V*(*s*_*j*_) = max_*l*_ (*Q*(*s*_*j*_, *a*_*l*_)). In the reduced task the second-step state value *V*(*s*_*j*_) was the value of the one action available in that state; *V*(*s*_*j*_) = *Q*(*s*_*j*_, *a*)

The action value update rule used by the model-based agent at the end of each trial was:

$$Q(s_{2},a_{2})\leftarrow \;(1-\alpha )Q(s_{2},a_{2})+\;\alpha \;r$$

In S5 Fig, a version of the model-based agent was used in which the transition probabilities *P*(*s*_*j*_|*a*_*i*_) linking the first-step actions to the second-step states was learnt online from experienced transitions. The update rule for the agents estimate of transition probabilities was:

$$\hat{P}\left(x|a_{1}\right)\leftarrow \left(1-\eta \right)\hat{P}\left(x|a_{1}\right)+\;\eta X$$

Where $\hat{P}\left(x|a_{1}\right)$ is the agents estimate of the probability of reaching state *x* ∈ (*a*, *b*) after choosing action *a*_1_, *η* is the transition probability learning rate, and *X* = 1 if the second-step state reached on the trial was *x* and *X* = 0 otherwise.

#### Agents with perseveration bias

In S6 Fig, versions of the Q(1) and model based agents with an additional perseveration bias were simulated on the reduced task. The perseveration bias was implemented as a transient increase to the value of the first-step action chosen on the previous trial. This boost to the action value was only used in determining choice probabilities and did not contribute to the action value update following the choice. The strength of perseveration bias used was 0.4.

#### Reward-as-cue agent

The reward-as-cue agent treated the choice between actions A and B as occurring in one of four different states on each trial, corresponding to the 4 combinations of the outcome (1 or 0) and second-step state (*a* or *b*) that occurred on the previous trial. The action value update used was:

$$Q(s_{1},a_{1})\leftarrow \;(1-\alpha )Q(s_{1},a_{1})+\;\alpha \;r$$

Where *Q*(*s*_1_, *a*_1_) is the value of the action chosen at the first step in the relevant state. In the original task where there was a choice at the second-step, action values for the second step actions were updated as:

$$Q(s_{2},a_{2})\leftarrow \;(1-\alpha )Q(s_{2},a_{2})+\;\alpha \;r$$

The reward as cue agent on the original task used separate softmax inverse temperatures and learning rates at the first and second steps.

#### Latent-state agent

The latent-state agent believed there were two states of the world, one of which had reward probabilities of (*P*_*good*_, *P*_*bad*_) in second-step states *a* and *b* respectively, and the other with reward probabilities of (*P*_*bad*_, *P*_*good*_) in second-step states *a* and *b* respectively. On the reduced task version *P*_*good*_ = 0.8 and *P*_*bad*_ = 0.2. On the original task version *P*_*good*_ = 0.625 and *P*_*bad*_ = 0.375.

At the start of each trial the agent performed a Bayesian update of the probability that the world was in each of these states based on the previous trial events. The agent then updated the probability that the world was in each state to account for the possibility that the world reversed in state between the previous and current trial, which was assumed to occur with probability ω. The agent used a probabilistic mapping from its estimate of the state of the world to choice, choosing with probability (1 – ε) the action with higher reward probability in the most probable state, and with probability ε the action with higher reward probability in the less probable state. On the original task where there was a choice at the second-step, action values for the second step actions were updated as:

$$Q(s_{2},a_{2})\leftarrow \;(1-\alpha )Q(s_{2},a_{2})+\;\alpha \;r$$

and the agent chose between the second-step actions using the softmax decision rule.

### Parameter values

The parameter values of the model-based agent on both tasks were set to: *α* = 0.5, *T* = 5

To ensure that average behaviour for the different agents was comparable, the parameters of the other agents were set by maximum likelihood fitting to data simulated from the model-based agent. This resulted in the following agent parameters:

**Table:** 

Original task:	
*Q*(0) agent:	*α* = 0.384, *T* = 4.09
*Q*(1) agent:	*α* = 0.398, *T* = 2.72
Reward-as-cue agent:	*α*_*first step*_=0.00184, *T*_*first step*_ = 4.82, *α*_*second step*_ = 0.499, *T*_*first step*_ = 4.98
Latent-state agent:	*ω* = 0.0882, ε = 0.368, *α* = 0.509, *T* = 4.96
Reduced task:	
*Q*(0) agent:	*α* = 0.501, *T* = 2.96
*Q*(1) agent:	*α* = 0.334, *T* = 3.22
*Q*(0.25) agent:	*α* = 0.499, *T*=3.48
*Q*(0.5) agent:	*α* = 0.477, *T*=3.57
*Q*(0.75) agent:	*α* = 0.409, *T* = 3.42
Reward-as-cue agent:	*α* = 0.00344, *T* = 4.46
Latent-state agent:	*ω* = 0.0326, ε = 0.188

### Comparing agent performance

To evaluate the performance of the different agents in Fig 4, agent parameter values were optimised using Powell’s method [55]. To reduce fluctuations in the objective function due to stochastic task and agent behaviour, the random seed was set to the same value at the start of every simulation in a given optimisation run. Each optimisation run was repeated 10 times from randomised initial parameter values to avoid local maxima. To prevent overestimation of performance due to overfitting to the specific pattern of behaviour generated by a given random seed, once parameters had been found which maximised performance for a given random seed, performance was evaluated with these parameters but a different random seed and this value was taken. For each agent, performance was evaluated for 10 sessions each of 10000 trials, with a different random seed used during the optimisation for each session. For those agents with only two parameters we separately optimised the performance using a brute force grid search. Performance evaluated using the Powell and grid search optimisation methods did not differ significantly for any agent. Values reported in the paper are from the Powell optimisation. For the Reward-as-cue agent we used the performance of a deterministic reward-as-cue strategy which choose option A following reward in state *a*, option B following reward in state *b*, choose option A following non-reward in state *b*, option B following non-reward in state *a*.

### Logistic regression analysis

In all logistic regression analyses, the dependent variable was the subject’s choice, coded as stay vs switch, such that positive values of the predictor promote staying with the previous choice. Predictors used in the analysis took the following values as a function of trial events:

*Stay*: +1 for all trials.

*Outcome*: +0.5 for rewarded trials, -0.5 for non-rewarded trials.

*Transition*: +0.5 for common transition trials, -0.5 for rare transition trials.

*Transition-outcome interaction*: +0.5 for common transition rewarded and rare transitions non-rewarded trials, -0.5 for rare transition rewarded and common transition non-rewarded trials.

*Correct—binary*: +0.5 for choosing option which led commonly to state with higher reward probability, -0.5 for choosing option which led commonly to state with lower reward probability. In the original task the higher reward probability of the two actions available in each second step state was taken as the states reward probability.

*Correct-continuous*: Difference between reward probability in the state commonly reached from the chosen action and reward probability in the state commonly reached from the not-chosen action.

*Rewarded-common*: +0.5 for rewarded trials with common transition, 0 otherwise.

*Rewarded-rare*: +0.5 for rewarded trials with rare transition, 0 otherwise.

*Non-rewarded common*: +0.5 for non-rewarded trials with common transition, 0 otherwise.

*Non-rewarded rare*: +0.5 for non-rewarded trials with rare transition, 0 otherwise.

## Supporting Information

S1 Fig Interaction predictor loading as a function of agent parameter values. Loading on the transition-outcome interaction predictor as a function of agent parameter values for behaviour simulated from different agent types on the reduced version of the task. Agent types: (**A**) *Q*(1), (**B**) *Q*(0), (**C**) Model-based, (**D**) Reward-as-cue, (**E**) Latent-state. The regression used predictors; stay, outcome, transition, transition-outcome interaction (as in Fig 3 centre left panels).
(EPS)

S2 Fig Reduced task with different reward probability distributions. (**A-E**) Simulation of *Q*(1) agent on version of reduced task with random walk reward probabilities. (**A**) Example of random walk reward probabilities in states *a* and *b*. (**B**) Stay probability plot. (**C**) Predictor loading for logistic regression analysis using predictors; stay, outcome, transition, transition-outcome interaction. (**D**) Predictor loadings for logistic regression using additional binary valued correct predictor. (**E**) Predictor loadings for logistic regression using additional continuous valued correct predictor. (**F**, **G**) Simulation of *Q*(1) agent on version of reduced task with fixed 0.2, 0.8 reward probabilities. (**H**, **I**) Simulation of *Q*(1) agent on version of reduced task with fixed 0.5, 0.5 reward probabilities.
(EPS)

S3 Fig Comparison of agents’ behaviour–original task. Comparison of the behaviour of all agents types discussed in the paper on the original task. Far left panels–Stay probability plots. Centre left panels—Predictor loadings for logistic regression model predicting whether the agent will repeat the same choice as a function predictors; stay, outcome, transition, transition-outcome interaction. Centre right panels–Predictor loadings for logistic regression analysis with additional ‘correct’ predictor. Right panels—Predictor loadings for lagged logistic regression model. Error bars in all plots show SEM across sessions. Agent types: (**A-D**) *Q*(1), (**E-H**) Model-based, (**I-L**) *Q*(0), (**M-P**) Reward-as-cue, (**Q-T**) Latent-state.
(EPS)

S4 Fig Intermediate values of lambda. Comparison of behaviour simulated on reduced task by agents with intermediate values of the lambda parameter that controls the relative contribution of the *Q*(1) update and TD0 update; (**A**-**D**) lambda = 0.25, (**E**-**H**) lambda = 0.5, (**I**-**L**) lambda = 0.75. Far left panels–Stay probability plots. Centre left panels—Predictor loadings for logistic regression model predicting whether the agent will repeat the same choice as a function predictors; stay, outcome, transition, transition-outcome interaction. Centre right panels–Predictor loadings for logistic regression analysis with additional ‘correct’ predictor. Right panels—Predictor loadings for lagged logistic regression model. Error bars in all plots show SEM across sessions.
(EPS)

S5 Fig Effect of transition matrix learning. Comparison of behaviour simulated on reduced task by model based agent that learned the transition probabilities online from the experienced transitions. (**A**-**D**) transition learning rate = 0.1, (**E**-**H**) transition learning rate = 0.5, (**I**-**L**) transition learning rate = 0.75. Far left panels–Stay probability plots. Centre left panels—Predictor loadings for logistic regression model predicting whether the agent will repeat the same choice as a function predictors; stay, outcome, transition, transition-outcome interaction. Centre right panels–Predictor loadings for logistic regression analysis with additional ‘correct’ predictor. Right panels—Predictor loadings for lagged logistic regression model. Error bars in all plots show SEM across sessions.
(EPS)

S6 Fig Effect of perseveration parameter. Comparison of behaviour simulated on reduced task by (**A**-**D**) *Q*(1) and (**E**-**H**) Model-based agents with an additional perseveration parameter which biases them to repeat choices. Far left panels–Stay probability plots. Centre left panels—Predictor loadings for logistic regression model predicting whether the agent will repeat the same choice as a function predictors; stay, outcome, transition, transition-outcome interaction. Centre right panels–Predictor loadings for logistic regression analysis with additional ‘correct’ predictor. Right panels—Predictor loadings for lagged logistic regression model. Error bars in all plots show SEM across sessions.
(EPS)

S7 Fig BIC score comparison–original task. BIC scores for maximum likelihood fits of different agent types (indicated by x-axis labels; MB–Model based, RC–Reward-as-cue, LS–Latent-state) to data simulated from each agent type (indicted by labels above axes; (**A**) *Q*(1) agent, (**B**) Q(0) agent, (**C**) Model-based agent, (**D**) Reward-as-cue agent, (**E**) Latent-state agent).
(EPS)

S1 Code Simulation code. (ZIP)
